# Regulation of human cerebrospinal fluid malate dehydrogenase 1 in sporadic Creutzfeldt-Jakob disease patients

**DOI:** 10.18632/aging.101101

**Published:** 2016-11-14

**Authors:** Matthias Schmitz, Franc Llorens, Alexander Pracht, Tobias Thom, Ângela Correia, Saima Zafar, Isidre Ferrer, Inga Zerr

**Affiliations:** ^1^ From the Department of Neurology, University Medical Center Göttingen, Göttingen, Germany; ^2^ German Center for Neurodegenerative Diseases (DZNE) – Göttingen Campus, Göttingen, Germany; ^3^ Institute of Neuropathology, Bellvitge University Hospital, CIBERNED, Hospitalet de Llobregat, University of Barcelona, Barcelona, Spain

**Keywords:** cerebrospinal fluid, diagnostic marker, Creutzfeldt-Jakob disease, PRNP codon 129 genotypes, mitochondrial malate dehydrogenase 1

## Abstract

The identification of reliable diagnostic biomarkers in differential diagnosis of neurodegenerative diseases is an ongoing topic. A previous two-dimensional proteomic study on cerebrospinal fluid (CSF) revealed an elevated level of an enzyme, mitochondrial malate dehydrogenase 1 (MDH1), in sporadic Creutzfeldt-Jakob disease (sCJD) patients. Here, we could demonstrate the expression of MDH1 in neurons as well as in the neuropil. Its levels are lower in sCJD brains than in control brains. An examination of CSF-MDH1 in sCJD patients by ELISA revealed a significant elevation of CSF-MDH1 levels in sCJD patients (independently from the *PRNP* codon 129 MV genotype or the prion protein scrapie (PrP^Sc^) type) in comparison to controls. In combination with total tau (tau), CSF-MDH1 detection exhibited a high diagnostic accuracy for sCJD diagnosis with a sensitivity of 97.5% and a specificity of 95.6%. A correlation study of MDH1 level in CSF with other neurodegenerative marker proteins revealed a significant positive correlation between MDH1 concentration with tau, 14-3-3 and neuron specific enolase level. In conclusion, our study indicated the potential of MDH1 in combination with tau as an additional biomarker in sCJD improving diagnostic accuracy of tau markedly.

## INTRODUCTION

Transmissible spongiform encephalopathies (TSE) belong to the group of neurodegenerative disorders, which are characterised by the conversion of the cellular prion protein (PrP^C^) to the disease-associated misfolded form PrPScrapie (PrP^Sc^). This group includes Creutzfeldt-Jakob disease (CJD), the most common form of prion disease in humans. CJD can be sporadic, genetic and acquired. All prion diseases are not curable with a rapid disease progression (survival time usually 6 months after disease onset).

The PrP gene (*PRNP*) encodes at codon 129 either the amino acid methionine (M) or valine (V). In the Caucasian population about 40% is homozygous for methionine, about 50% methionine/valine heterozygous, and about 10% homozygous for valine. The *PRNP* codon 129 polymorphism influences the conformational structure of the cellular PrP (PrP^C^) [1] and it may influence the disease course, symptoms at onset, type of the pathological changes such as the PrP^Sc^ deposition pattern and the sensitivity of diagnostic tests [2–5].

Although the diagnostics of sCJD has been improved markedly during the recent years, autopsies are still necessary for a confirmed diagnosis. All biomarkers which are currently available, such as 14-3-3, total tau (tau), S-100 calcium-binding protein B (S-100B), alpha-synuclein or neuron specific enolase (NSE) and the recently established *in vitro* amplification assays real time quaking induced conversion (RT-QuIC), have a relative high accuracy but their sensitivities depending from the *PRNP* codon 129 MV genotype are ranging between 80-90%, which may result in false negative diagnoses [6–15].

In this context, a previous high-throughput two-dimensional proteomic approach on sCJD brain homogenates and CSF in our group indicated a regulation of mitochondrial malate dehydrogenase 1 (MDH1), an enzyme that reversibly catalyses the oxidation of malate to oxaloacetate (part of many metabolic pathways, including the citric acid cycle) [16], in sCJD patients compared to controls [17, 18].

These findings prompted us to measure the concentration of MDH1 in brain tissue and CSF of sCJD patients (different geno- and subtypes) in comparison to control cases. Moreover, we analysed the diagnostic accuracy of MDH1 alone and in combination with surrogate biomarkers. To estimate the association of MDH1 level with other neurodegenerative marker proteins, we additionally performed a correlation analysis between MDH1 and tau, p-tau, 14-3-3, S-100B, NSE, Aβ1-40, Aβ1-42 and the Aβ ratio.

## RESULTS

### Analysis of MDH1 expression and localization in sCJD brain tissue

**Figure 1:**
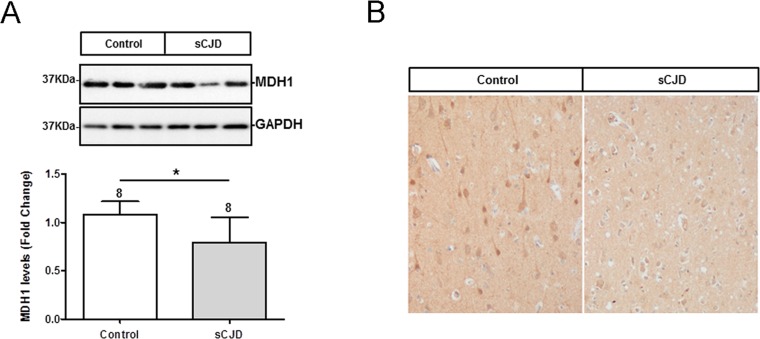
Determination of expression level and localization of MDH1 in brain tissue of CJD patients (**A**) MDH1 was detected by western blot analysis in the frontal cortex region 8 of sCJD patients (n=8) in comparison to controls (n=8). Densitometric analysis of band intensities revealed a lower of MDH1 level in sCJD patients (MM1 and VV2). (**B**) Immunohistochemical staining using anti-MDH1 confirmed the decreased expression of MDH1 in sCJD (MM1) patients (n=5) compared to age-matched controls (n=4). For comparison between two groups we used the *Wilcoxon Mann Whitney* test as appropriate. A p-value < 0.001 is considered as extremely significant (***), < 0.01 as very significant (**), < 0.05 as significant (*) and ≥ 0.05 as not significant (ns).

The level of MDH1 in the frontal cortex region of sCJD (n=8) patients was analysed by western blotting. MDH1 signal was quantified by using the software *Image J software*. Interestingly, we found levels of MDH1 reduced in brain tissue of sCJD patients compared to control brains (from patients without prion disease) (Fig. 1A).

Immunohistochemical staining of cytosolic MDH1 was performed in the frontal cortex region of sCJD (MM1) patients in comparison to non-prion disease controls. We observed MDH1 expressed in neurons as well as in the neuropil. The intensity of MDH1 staining was lower in sCJD cases than in controls, which was in agreement with the western blot observations (Fig. 1B).

### Determination of CSF-MDH1 levels in sCJD patients

**Figure 2:**
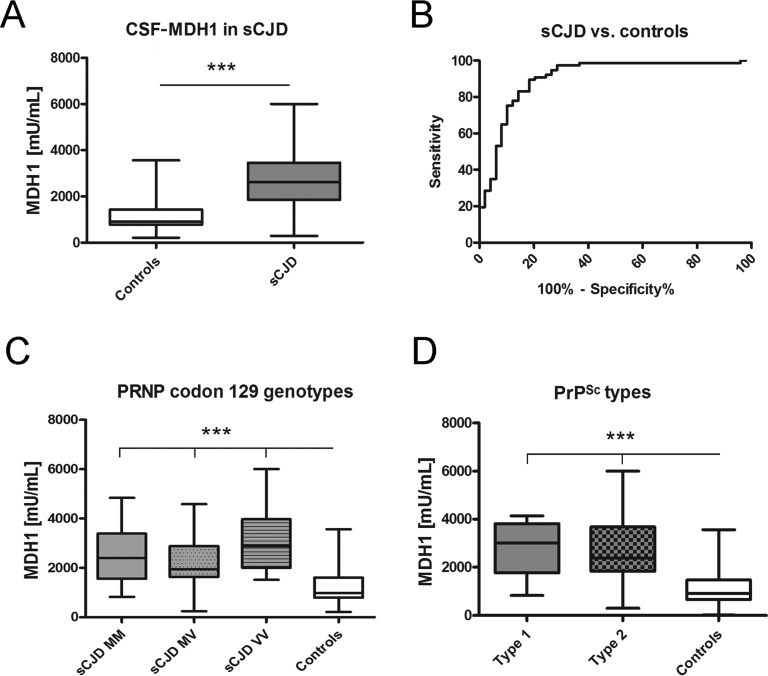
Determination of MDH1 levels in CSF of sCJD patients MDH1 was quantified by ELISA in CSF of sCJD and control patients. (**A**) Patients with sCJD (n=82) exhibited a significantly increased level of MDH1 compared to controls (n=60). (**B**) ROC curve analysis was performed for the discrimination between sCJD patients and controls. (**C**-**D**) MDH1 analysis of different *PRNP* codon 129 genotypes and PrP^Sc^ types in sCJD patients revealed no significant differences within the sCJD cohort (only when compared to controls). AUC values, corresponding to the area under ROC curves, and 95% confidence intervals are reported. For comparison between groups we used *one-way ANOVA or Wilcoxon Mann Whitney and Tukey's post hoc* test. A p-value < 0.001 is considered as extremely significant (***), < 0.01 as very significant (**), < 0.05 as significant (*) and ≥ 0.05 as not significant (ns).

At first, we investigated the levels of MDH1 by ELISA (indicated as million units per mL (mU/mL)) in CSF of sCJD patients in comparison to control cases without prion disease. The obtained data revealed a statistically significant difference between sCJD and controls (p<0.001) indicating that CSF-MDH1 is specifically increased in sCJD patients (Fig. 2A). The discrimination accuracy between sCJD and controls was 0.91 AUC (p<0.0001). Setting a cut off at 1530 mU/mL (defined by *Youden* index), the test exhibited a specificity of 85.0% and a sensitivity of 82.9% (Fig. 2B, Table 1). When we stratified our sCJD cohort according to three *PRNP* codon 129 genotypes (MM, MV, VV) as well as to PrP^Sc^ type 1 and 2, we observed no significant effect of the *PRNP* genotype or the type of PrP^Sc^ on the MDH1 level (Fig. 2C, D).

**Table 1:** Sensitivity and specificity of a single marker and in combination Sensitivity and specificity was calculated for MDH1, 14-3-3 and tau from the numbers of correctly classified samples. The combination of MDH1 and tau revealed the highest diagnostic accuracy.

Patients (n=82)	MDH1 (>1530 mU/mL)	Tau (>1300 pg/mL)	14-3-3 (pos./neg.)	MDH1 and 14-3-3	MDH1 and tau	14-3-3 and tau
sCJD	68/82 82.9 % sensitivity	73/82 89.0% sensitivity	70/82 85.4% sensitivity	75/82 91.4% sensitivity	80/82 97.5% sensitivity	74/82 90.2% sensitivity
Controls	51/60 85.0 % specificity	29/37* 78.4% specificity	30/37* 81.1% specificity	34/37* 91.9% specificity	35/37* 95.6% specificity	33/37* 89.2% specificity

* Only samples were considered where MDH1, Tau and 14-3-3 have been determined

### Analysis of diagnostic accuracy of MDH1 in comparison to 14-3-3 and tau as well as in combination

At first, we determined the diagnostic values of both neurodegenerative markers, 14-3-3 and tau, in comparison to MDH1. Both markers revealed a slightly higher sensitivity and a lower specificity than MDH1 (Table 1). As reported before, a combination of different biomarker proteins may improve the diagnostic value and reliability of biomarker detection [11, 27, 28]. Therefore, we explored if the combination of MDH1/14-3-3, MDH1/tau and 14-3-3/tau may increase the diagnostic accuracy. Interestingly, the combination of MDH1 with tau revealed the highest number of properly classified patients exhibiting a sensitivity of 97.5% and a specificity of 95.6% compared to others combinations (Table 1).

### Correlation between MDH1 and other neurodegenerative markers

**Figure 3:**
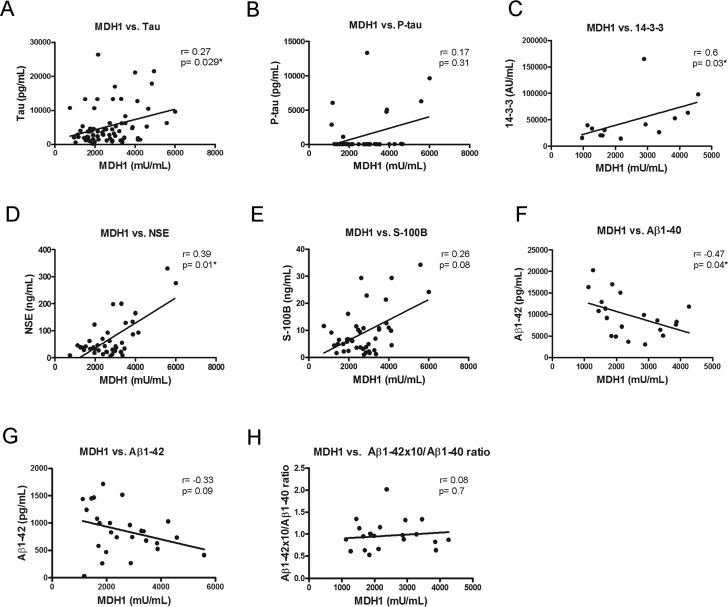
Correlation between MDH1 and neurodegenerative biomarker proteins In sCJD patients, a positive correlation was obtained between MDH1 level and the tau (r=0.27; p=0.029*; n=72), 14-3-3 (r=0.60; p=0.03*; n=13), NSE (r=0.39; P=0.01*; n=40) levels (**A**, **C**, **D**). MDH1 levels concentrations correlated negatively to Aβ1-40 (r=-0.47, p=0.04*, n=20) (**F**). No correlation was detected between MDH1 and p-tau (r=0.17, p=0.31, n=38), S-100B (r=0.26; p=0.08; n=43), Aβ1-42 (r=-0.33, p=0.09, n=26) levels and the Aβ1-42/Aβ1-40 ratio (r=0.08, p=0.7, n=20) (**B**, **E**, **G**, **H**). All correlation studies were computed by using the non-parametric *Spearman* correlation test (two-tailed) in a confidence interval of 95%. A p-value < 0.001 is considered as extremely significant (***), < 0.01 as very significant (**), < 0.05 as significant (*) and ≥ 0.05 as not significant (ns).

To examine a potential association between MDH1 and other already known neurodegenerative biomarker proteins, we initiated a correlation study. All marker concentrations were determined in the same patients. Interestingly, we observed in sCJD patients a positive relationship between MDH1 concentration and tau, 14-3-3 and NSE levels (Fig. 3A, C, D) while a negative correlation was found between MDH1 concentration and Aβ1-40 level (Fig. 3F). In contrast, no correlation was observed between MDH1 concentration and p-tau-, S-100B, Aβ1-42 level and Aβ1-42/1-40 ratio (Fig.3B, E, G, H). Levels of MDH1 were unrelated to age or gender (p>0.05).

## DISCUSSION

In the prion disease field, the need for an improved diagnostic accuracy requires the identification of novel biomarkers. Even though already known biomarkers such as 14-3-3 and tau or the detection of PrP^Sc^ by RT-QuIC has exhibited a high diagnostic accuracy to discriminate CJD from control cases [11, 15, 28], there is no marker available which shows a sensitivity close to 100%. Most markers are described with a sensitivity varying between 80-90% [9, 11, 15, 28, 29].

A previously conducted two-dimensional proteomic study on CJD brain homogenates and CSF indicated a specific regulation of MDH1 in sCJD patients compared to controls [17, 18].

We could confirm the decrease of MDH1 in the frontal cortex brain region of sCJD patients by western blotting and by immunohistochemical analysis which further revealed the expression of MDH1 in neurons as well as in the neuropil. Since MDH1 is involved in the energy metabolism our observations fits to previous data indicating that the energetic metabolism is altered in sCJD [30].

This very interesting finding encouraged us to study the MDH1 level in CSF of sCJD patients, in comparison to control cases without prion disease.

Our objectives were to evaluate the potential of MDH1 to discriminate sCJD patients from controls as well as to analyse the influence of the *PRNP*-codon 129 MV genotype and the PrP^Sc^ type on the CSF level of MDH1. Interestingly, we observed an inverse relationship between MDH1 level in brain and CSF. An explanation may be that cytosolic neuronal MDH1 is released into the extracellular space as a consequence of neuronal damage. Therefore, lower levels are detectable in post-mortem sCJD brains, where massive neuronal damage has already occurred. As a consequence of neuronal damage, MDH1 can be filtered to the CSF where elevated levels can be detected. This is in agreement with similar brain-CSF correlations detected for other sCJD surrogate markers such as 14-3-3 and tau [31].

For diagnostic discrimination, the higher MDH1 level in CSF of sCJD patients (compared to controls) revealed a specificity of 85.0% and a sensitivity of 82.9%. Com-pared to that, 14-3-3 and tau exhibited sensitivity values of 85.4% and 89.0%, which is in line with others [9, 11, 28, 29] and slightly higher than the sensitivity of MDH1.

However, when we examined the sensitivities of different biomarkers in combination, we observed that the MDH1/tau combination revealed the best diagnostic accuracy with a sensitivity of 97.5% and a specificity of 95.6%. In this context, Sanchez-Juan et al., have already reported that a combined determination of 14-3-3 and tau, S-100B, or NSE increases the sensitivity to over 93% for sCJD [28], which highlights the importance of a combinative biomarker analysis. Variations among different studies may be explained by composition of the patient cohort different test systems, or by demographical differences.

When we analysed the impact of the *PRNP* codon 129 MV polymorphism and the PrP^Sc^ type on MDH1 levels, both influence clinical and pathological phenotype of sCJD [2–5], we observed that the MDH1 concentration did not depend on either of these factors. Other diagnostic biomarker or assays, such as 14-3-3 or RT-QuIC also failed to distinguish between different sCJD genotypes or PrP^Sc^ type 1 and 2 [32, 33]. In contrast, tau can be used to assess the PrP^Sc^ type in sCJD cases but not *PRNP* codon 129 genotype [34].

Correlation studies of MDH1 levels with several neurodegenerative marker proteins such as tau, 14-3-3 and NSE revealed significant correlations between the MDH1 and these marker proteins, which indicate a dependence of MDH1 levels to neuronal damage in brains of sCJD patients. It can be speculated whether neuronal MDH1 also reflects the extent of neuronal damage in the brain tissue.

For a further evaluation of MDH1 as an additional marker in sCJD diagnosis as well as in combination with tau, present findings must be validated in a larger patient cohort and the reproducibility of the test needs to be proven by ring trial studies. Moreover, a potential association of MDH1 with the disease stage or the duration of the disease needs to be investigated further.

In conclusion, the present study has suggested CSF-MDH1 as a potential novel diagnostic biomarker in sCJD, in particular the combination of MDH1 and tau improved the sensitivity of both markers markedly.

## MATERIALS AND METHODS

### Patients

The cohort of patients in this study consisted of 82 sCJD (44 male, 38 female), (26xMM, 26xMV and 30xVV) aged 53-83 years; mean age 64.9 ± 8.5 years.

Thirty five patients were classified as definite cases by neuropathological examinations, the rest as probable CJD cases according to diagnostic consensus criteria [19–22].

Age and gender matched control cases (n=60) are patients with an either clinically or pathologically defined alternative diagnosis, such as psychiatric disorders, dizziness, headache, vasculitis, encephalitis, viral meningitis, neuroborreliosis, Parkinson's disease, vascular dementia, Alzheimer's disease and normal-pressure hydrocephalus.

After collection, CSF samples were centrifuged for 10 min at 720 x g (Eppendorf model no. 5415C centrifuge) at room temperature (~22°C), aliquoted in 1 mL portions and stored at −80°C. Blood-stained CSF samples were excluded from the study and we avoided thawing and refreezing the same samples more than 8 times.

The analysis of the codon 129 genotype of *PRNP* was performed after isolation of genomic DNA from blood samples according to standard methods [23].

### Determination of human malate dehydrogenase 1

CSF levels of human MDH1 were measured using a commercially available ELISA kit (Cusabio, Hubei, China). We followed the manufacturer's instructions. Briefly, levels of MDH1 were measured in million units (mU) per mL. The detection range of the assay reaches from 9.38 mU/mL to 600 mU/mL. To be in this range, CSF samples were measured in a dilution of 1:100 using the sample diluent buffer supplied in the kit. Colorimetric reaction was measured at 450 nm with a 1420 Multilabel Counter Victor 2 (Wallac) (PerkinElmer, Massachusetts, USA).

### Determination of 14-3-3 protein gamma in CSF by ELISA

The amount of 14-3-3 gamma protein in CSF was ascertained by using the *CircuLex 14-3-3 Gamma ELISA Kit* (BIOZOL Diagnostica Vertrieb GmbH, Eching, Germany) by following the manufacturer's instructions and as described before [11]. Briefly, CSF samples were applied in a dilution of 1:5 in dilution buffer. The level of 14-3-3 gamma was measured in arbitrary units (AU) per mL (1 AU is almost equal to 1 pg). For capturing, CSF samples were incubated for one hour at room temperature. After washing, 14-3-3 gamma detection antibody and later horseradish peroxidase-conjugated anti-IgG were added and incubated for one hour at room temperature each. Colorimetric reaction was measured at 450 nm using a 1420 Multilabel Counter Victor 2 Wallac) (PerkinElmer, Massachusetts, USA).

### Determination of Aβ1-40 and Aβ1-42 levels in CSF

Levels of Aβ1-40 were measured by using the full-length Aβ1-40 high sensitive assay ELISA obtained from IBL (Hamburg, Germany) with a detection range between 1.56-100 pg/mL. Levels of Aβ1-42 were ascertained by the use of an ELISA kit named INNOTEST®-AMYLOID (1–42) (Innogenetics, Hannover, Germany) with a detection range between 125-2000 pg/mL. All ELISA measurements were performed according to the protocol of the manufacturer.

### Determination of tau and p-tau 181 levels in CSF

CSF levels of tau were determined by using an ELISA kit from INNOTEST® hTAU Ag (Innogenetics) with a detection range between 50-2500 pg/mL (detection limit: 34 pg/mL). Concentration of phosphorylated tau at threonine-181 (p-tau) was measured by using an ELISA kit named INNOTEST®PHOSPHO-TAU (181 P) (Inno-genetics) with a detection range between 15.6-1000 pg/mL (detection limit: 3 pg/mL).

Briefly, before antibody incubations, each sample (75 μl) was diluted 1:1 in sample diluent. The colorimetric reaction was measured at 450 nm with a 1420 Multilabel Counter Victor 2 (Wallac) (PerkinElmer). Each sample was measured in duplicate. For analysis we calculated the median.

### SDS-PAGE and immunoblotting

For Western blot analysis we followed a modified protocol as described previously [24, 25]. A monoclonal MDH1 antibody (Acris-Antibodies GmbH, Herford, Germany) was diluted 1:1000. As a secondary antibody we used a polyclonal horseradish peroxidase-conjugated antibody (Jackson Immuno Research, Leipzig, Germany).

For denaturation samples were heated for 2 min at 95°C, after 10 μL sample loading buffer (Bio-Rad Laboratories GmbH, Munich, Germany) had been added. Protein separation occurred by sodium dodecyl sulphate-polyacrylamide gel electrophoresis (SDS-PAGE) (12% w/v polyacrylamide). Subsequently, proteins were transferred to polyvinylidene difluoride (PVDF) Hydrobond-P membranes (Amersham, Freiburg, Germany) using a semi-dry transblot cell. After blocking with 5% dried milk in PBS and 0.1% Tween-20 for 1 h at room temperature, samples were incubated with the primary antibody overnight at 4°C. Membranes were rinsed in PBS-Tween and incubated with the corresponding horseradish peroxidase-conjugated secondary antibody (Jackson Immuno Research, Leipzig, Germany) (diluted 1:10000). Protein bands were visualized after immersion of the membranes in enhanced chemiluminescence (ECL) detection system solution using Chemicon system (Bio-Rad Laboratories GmbH). The western blot bands obtained from 8 control- and 8 sCJD cases (frontal cortex region 8) were quantified by densitometry using *Image J software*. MDH1 levels were normalized with those obtained from the loading control protein glyceraldehyde 3-phosphate dehydrogenase (GAPDH).

### Immunohistochemistry

Brain samples (frontal cortex region 8) from sCJD MM1 patients (n= 5) and aged matched controls (n= 4) were treated with formic acid and were embedded in paraffin. Sections, 4 microns thick, were treated with citrate buffer (20 min) to enhance antigenicity. Endogenous peroxidases were blocked with 10% methanol and 1% H_2_O_2_ (15 min) followed by 3% normal horse serum and then incubated at 4°C overnight with the primary antibody used at a dilution of 1:100. Immediately afterwards the sections were incubated with EnVision Systems (Dako Deutschland GmbH, Hamburg, Germany) at room temperature (15 min). The peroxidase reaction was visualized with diamino-benzidine and H_2_O_2_. The omission of the primary antibody in some sections was used as control of the immunostaining; no signal was obtained with the only incubation being the secondary antibody.

### Ethics

The study was conducted according to the revised Declaration of Helsinki and Good Clinical Practice guidelines. It has been approved by the local ethic committee at the University Medical Center Göttingen (Goettingen, Germany) (No. 11/11/93). Informed consent was given by all study participants or their legal next of kin.

### Statistical analysis

Statistical data analysis was conducted by the use of the statistic software *GraphPath Prism* 6.01. The *Mann-Whitney* test was used for comparison between two groups. For comparison between more than two groups, we used the *One-way ANOVA* test followed by *Tukey* post hoc analysis. All correlation studies were computed by using the non-parametric *Spearman* correlation test (two-tailed) in a confidence interval of 95%. P-values below 0.05 are considered as significant. Optimal cut off values were estimated based on the *Youden index* [26].

## Abbreviations

Aβ: Amyloid beta;
AUC: relative area under the curve
CJD: Creutzfeldt-Jakob disease
sCJD: sporadic CJD
CSF: cerebrospinal fluid
MDH1: mitochondrial malate dehydrogenase 1
NSE: neuron specific enolase
PrP^C^: cellular prion protein
PrP^Sc^: scrapie prion protein
ROC: receiver operating characteristic
rpm: revolutions per minute
RT-QuIC: real-time quaking-induced conversion
SEM: standard error of the mean
S-100B: S-100 calcium-binding protein B
TSE: transmissible spongiforme encephalopathies, total tau, tau

## References

[1] Pham N, Yin S, Yu S, Wong P, Kang SC, Li C, Sy MS (2008). Normal cellular prion protein with a methionine at position 129 has a more exposed helix 1 and is more prone to aggregate. Biochem Biophys Res Commun.

[2] Collins SJ, Sanchez-Juan P, Masters CL, Klug GM, van Duijn C, Poleggi A, Pocchiari M, Almonti S, Cuadrado-Corrales N, de Pedro-Cuesta J, Budka H, Gelpi E, Glatzel M (2006). Determinants of diagnostic investigation sensitivities across the clinical spectrum of sporadic Creutzfeldt-Jakob disease. Brain.

[3] Krasnianski A, Meissner B, Schulz-Schaeffer W, Kallenberg K, Bartl M, Heinemann U, Varges D, Kretzschmar HA, Zerr I (2006). Clinical features and diagnosis of the MM2 cortical subtype of sporadic Creutzfeldt-Jakob disease. Arch Neurol.

[4] Meissner B, Westner IM, Kallenberg K, Krasnianski A, Bartl M, Varges D, Bösenberg C, Kretzschmar HA, Knauth M, Schulz-Schaeffer WJ, Zerr I (2005). Sporadic Creutzfeldt-Jakob disease: clinical and diagnostic characteristics of the rare VV1 type. Neurology.

[5] Zerr I, Pocchiari M, Collins S, Brandel JP, de Pedro Cuesta J, Knight RS, Bernheimer H, Cardone F, Delasnerie-Lauprêtre N, Cuadrado Corrales N, Ladogana A, Bodemer M, Fletcher A (2000). Analysis of EEG and CSF 14-3-3 proteins as aids to the diagnosis of Creutzfeldt-Jakob disease. Neurology.

[6] Zerr I, Bodemer M, Räcker S, Grosche S, Poser S, Kretzschmar HA, Weber T (1995). Cerebrospinal fluid concentration of neuron-specific enolase in diagnosis of Creutzfeldt-Jakob disease. Lancet.

[7] Beaudry P, Cohen P, Brandel JP, Delasnerie-Lauprêtre N, Richard S, Launay JM, Laplanche JL (1999). 14-3-3 protein neuron-specific enolase and S-100 protein in cerebrospinal fluid of patients with Creutzfeldt-Jakob disease. Dement Geriatr Cogn Disord.

[8] Otto M, Stein H, Szudra A, Zerr I, Bodemer M, Kretzschmar HA, Mäder W, Poser S, Weber T (1996). Elevated level of S100 protein concentration in CSF of patients with Creutzfeldt-Jakob disease. Aktuelle Neurol.

[9] Otto M, Wiltfang J, Cepek L, Neumann M, Mollenhauer B, Steinacker P, Ciesielczyk B, Schulz-Schaeffer W, Kretzschmar HA, Poser S (2002). Tau protein and 14-3-3 protein in the differential diagnosis of Creutzfeldt-Jakob disease. Neurology.

[10] Kohira I, Tsuji T, Ishizu H, Takao Y, Wake A, Abe K, Kuroda S (2000). Elevation of neuron-specific enolase in serum and cerebrospinal fluid of early stage Creutzfeldt-Jakob disease. Acta Neurol Scand.

[11] Schmitz M, Ebert E, Stoeck K, Karch A, Collins S, Calero M, Sklaviadis T, Laplanche JL, Golanska E, Baldeiras I, Satoh K, Sanchez-Valle R, Ladogana A (2016). Validation of 14-3-3 protein as a marker in sporadic Creutzfeldt-Jakob disease diagnostic. Mol Neurobiol.

[12] Atarashi R, Satoh K, Sano K, Fuse T, Yamaguchi N, Ishibashi D, Matsubara T, Nakagaki T, Yamanaka H, Shirabe S, Yamada M, Mizusawa H, Kitamoto T (2011). Ultrasensitive human prion detection in cerebrospinal fluid by real-time quaking-induced conversion. Nat Med.

[13] McGuire LI, Peden AH, Orrú CD, Wilham JM, Appleford NE, Mallinson G, Andrews M, Head MW, Caughey B, Will RG, Knight RS, Green AJ (2012). Real time quaking-induced conversion analysis of cerebrospinal fluid in sporadic Creutzfeldt-Jakob disease. Ann Neurol.

[14] Llorens F, Kruse N, Schmitz M, Shafiq M, da Cunha JE, Gotzman N, Zafar S, Thüne K, de Oliveira JR, Mollenhauer B, Zerr I (2015). Quantification of CSF biomarkers using an electrochemiluminescence-based detection system in the differential diagnosis of AD and sCJD. J Neurol.

[15] Cramm M, Schmitz M, Karch A, Mitrova E, Kuhn F, Schroeder B, Raeber A, Varges D, Kim YS, Satoh K, Collins S, Zerr I (2016). Stability and reproducibility underscore utility of RT-QuIC for diagnosis of Creutzfeldt-Jakob disease. Mol Neurobiol.

[16] Minárik P, Tomásková N, Kollárová M, Antalík M (2002). Malate dehydrogenases--structure and function. Gen Physiol Biophys.

[17] Gawinecka J, Cardone F, Asif AR, De Pascalis A, Wemheuer WM, Schulz-Schaeffer WJ, Pocchiari M, Zerr I (2012). Sporadic Creutzfeldt-Jakob disease subtype-specific alterations of the brain proteome: impact on Rab3a recycling. Proteomics.

[18] Gawinecka J, Dieks J, Asif AR, Carimalo J, Heinemann U, Streich JH, Dihazi H, Schulz-Schaeffer W, Zerr I (2010). Codon 129 polymorphism specific cerebrospinal fluid proteome pattern in sporadic Creutzfeldt-Jakob disea-. se and the implication of glycolytic enzymes in prion-induced pathology. J Proteome Res.

[19] WHO (1998). Human transmissible spongiform encephalo-pathies. Wkly Epidemiol Rec.

[20] WHO (2003). Diagnostic tests for human transmissible spongiform encephalopathies WHO Manual for Surveillance of Human Transmissible Spongiform Encephalopathies including variant Creutzfeldt-Jakob disease.

[21] Zerr I, Schulz-Schaeffer WJ, Giese A, Bodemer M, Schröter A, Henkel K, Tschampa HJ, Windl O, Pfahlberg A, Steinhoff BJ, Gefeller O. A. KH, Poser S (2000). Current clinical diagnosis in CJD: identification of uncommon variants. Ann Neurol.

[22] Zerr I, Kallenberg K, Summers DM, Romero C, Taratuto A, Heinemann U, Breithaupt M, Varges D, Meissner B, Ladogana A, Schuur M, Haik S, Collins SJ (2009). Updated clinical diagnostic criteria for sporadic Creutzfeldt-Jakob disease. Brain.

[23] Windl O, Giese A, Schulz-Schaeffer W, Zerr I, Skworc K, Arendt S, Oberdieck C, Bodemer M, Poser S, Kretzschmar HA (1999). Molecular genetics of human prion diseases in Germany. Hum Genet.

[24] Schmitz M, Schlomm M, Hasan B, Beekes M, Mitrova E, Korth C, Breil A, Carimalo J, Gawinecka J, Varges D, Zerr I (2010). Codon 129 polymorphism and the E200K mutation do not affect the cellular prion protein isoform composition in the cerebrospinal fluid from patients with Creutzfeldt-Jakob disease. Eur J Neurosci.

[25] Schmitz M, Lüllmann K, Zafar S, Ebert E, Wohlhage M, Oikonomou P, Schlomm M, Mitrova E, Beekes M, Zerr I (2014). Association of prion protein genotype and scrapie prion protein type with cellular prion protein charge isoform profiles in cerebrospinal fluid of humans with sporadic or familial prion diseases. Neurobiol Aging.

[26] Youden WJ (1950). Index for rating diagnostic tests. Cancer.

[27] Llorens F, Schmitz M, Karch A, Cramm M, Lange P, Gherib K, Varges D, Schmidt C, Zerr I, Stoeck K (2016). Comparative analysis of cerebrospinal fluid biomarkers in the differential diagnosis of neuro-degenerative dementia. Alzheimers Dement.

[28] Sanchez-Juan P, Green A, Ladogana A, Cuadrado-Corrales N, Sáanchez-Valle R, Mitrováa E, Stoeck K, Sklaviadis T, Kulczycki J, Hess K, Bodemer M, Slivarichová D, Saiz A (2006). CSF tests in the differential diagnosis of Creutzfeldt-Jakob disease. Neurology.

[29] Chohan G, Pennington C, Mackenzie JM, Andrews M, Everington D, Will RG, Knight RS, Green AJ (2010). The role of cerebrospinal fluid 14-3-3 and other proteins in the diagnosis of sporadic Creutzfeldt-Jakob disease in the UK: a 10-year review. J Neurol Neurosurg Psychiatry.

[30] Ansoleaga B, Garcia-Esparcia P, Llorens F, Hernández-Ortega K, Carmona Tech M, Antonio Del Rio J, Zerr I, Ferrer I (2016nlw048). Altered Mitochondria Protein Synthesis Machinery and Purine Metabolism Are Molecular Contributors to the Pathogenesis of Creutzfeldt-Jakob Disease. J Neuropathol Exp Neurol.

[31] Llorens F, Zafar S, Ansoleaga B, Shafiq M, Blanco R, Carmona M, Grau-Rivera O, Nos C, Gelpí E, Del Río JA, Zerr I, Ferrer I (2015). Subtype and regional regulation of prion biomarkers in sporadic Creutzfeldt-Jakob disease. Neuropathol Appl Neurobiol.

[32] Gmitterová K, Heinemann U, Bodemer M, Krasnianski A, Meissner B, Kretzschmar HA, Zerr I (2009). 14-3-3 CSF levels in sporadic Creutzfeldt-Jakob disease differ across molecular subtypes. Neurobiol Aging.

[33] Cramm M, Schmitz M, Karch A, Zafar S, Varges D, Mitrova E, Schroeder B, Raeber A, Kuhn F, Zerr I (2015). Characteristic CSF prion seeding efficiency in humans with prion diseases. Mol Neurobiol.

[34] Karch A, Hermann P, Ponto C, Schmitz M, Arora A, Zafar S, Llorens F, Müller-Heine A, Zerr I (2015). Cerebrospinal fluid tau levels are a marker for molecular subtype in sporadic Creutzfeldt-Jakob disease. Neurobiol Aging.

